# Adaptive Admittance Control for an Ankle Exoskeleton Using an EMG-Driven Musculoskeletal Model

**DOI:** 10.3389/fnbot.2018.00016

**Published:** 2018-04-10

**Authors:** Shaowei Yao, Yu Zhuang, Zhijun Li, Rong Song

**Affiliations:** ^1^Key Laboratory of Sensing Technology, Biomedical Instrument of Guangdong Province, School of Engineering, Sun Yat-sen University, Guangzhou, China; ^2^Key Laboratory of Autonomous System and Network Control, College of Automation Science and Engineering, South China University of Technology, Guangzhou, China

**Keywords:** joint stiffness, musculoskeletal model, rehabilitation robot, robot control, EMG

## Abstract

Various rehabilitation robots have been employed to recover the motor function of stroke patients. To improve the effect of rehabilitation, robots should promote patient participation and provide compliant assistance. This paper proposes an adaptive admittance control scheme (AACS) consisting of an admittance filter, inner position controller, and electromyography (EMG)-driven musculoskeletal model (EDMM). The admittance filter generates the subject's intended motion according to the joint torque estimated by the EDMM. The inner position controller tracks the intended motion, and its parameters are adjusted according to the estimated joint stiffness. Eight healthy subjects were instructed to wear the ankle exoskeleton robot, and they completed a series of sinusoidal tracking tasks involving ankle dorsiflexion and plantarflexion. The robot was controlled by the AACS and a non-adaptive admittance control scheme (NAACS) at four fixed parameter levels. The tracking performance was evaluated using the jerk value, position error, interaction torque, and EMG levels of the tibialis anterior (TA) and gastrocnemius (GAS). For the NAACS, the jerk value and position error increased with the parameter levels, and the interaction torque and EMG levels of the TA tended to decrease. In contrast, the AACS could maintain a moderate jerk value, position error, interaction torque, and TA EMG level. These results demonstrate that the AACS achieves a good tradeoff between accurate tracking and compliant assistance because it can produce a real-time response to stiffness changes in the ankle joint. The AACS can alleviate the conflict between accurate tracking and compliant assistance and has potential for application in robot-assisted rehabilitation.

## Introduction

Many studies have shown that robot-aided rehabilitation training can effectively improve the motor functions of stroke patients (Song et al., 2008, 2013). Robots can ensure the consistency of repetitive and intense therapeutic interventions. Thus, various robot-aided devices have been developed and widely used to help physical therapists in interactive training for the physically disabled (Jamwal et al., 2016; Ayas and Altas, 2017).

Patient participation is important in facilitating neuromuscular recovery during robot-aided rehabilitation (Kleim and Jones, 2008; Ao et al., 2017). Moreover, patient participation is related to the control schemes used in robots. For instance, a proportional-derivative (PD) position control scheme is widely utilized for trajectory tracking. In this control scheme, patients passively participate in human-robot cooperative movement, but the human-robot interaction has not been taken into account (Meng et al., 2015).

To promote patient participation, assist-as-needed control schemes have been developed. The impedance control proposed by Hogan (1985) has been used in robot-assisted rehabilitation because it can regulate the desired dynamic relationship between the robot end-effector position and the interaction force in real time. Jamwal et al. (2016) designed three different compliance levels of impedance control for an ankle rehabilitation robot and found that increasing the robotic compliance could encourage the subjects to participate more actively in the training process owing to appropriate deviation from the desired path. In addition, a compliant force field was proposed and designed to achieve an assist-as-needed control scheme (Srivastava et al., 2015; Wu et al., 2017). Despite these innovations, patients still need to follow the predefined trajectory in rehabilitation training.

Recently, there has been an increasing tendency to apply admittance control in exoskeletons to allow compliant human-robot interaction without a pre-defined trajectory (Aguirre-Ollinger et al., 2016; Ayas and Altas, 2017). This control scheme consists of two parts, namely, an admittance filter and an inner position loop. The admittance filter is applied to generate the desired position according to the user's joint torque (Saglia et al., 2013). Then the assistive torque is determined by the inner position loop using its parameter settings and the desired position. Therefore, the quality of the inner position loop greatly influences the performance and stability of admittance control (Pelletier and Doyon, 1994) because the loop can compensate for the effects of unmodeled factors, such as friction (Bruno and Oussama, 2008). In a previous study, admittance control was considered robust in the inner loop when well-behaved position control was used (Calanca et al., 2015). However, the parameters of the loop should not be maintained at a constant level because this does not account for changes in the patient's limb condition.

Electromyography (EMG) signals contain rich information about muscle activity and voluntary movement, so it can be used in robot-assisted rehabilitation to enhance voluntary participation. Song et al. (2013) developed a myoelectrically controlled robotic system to provide continuous stretching-assistance torque that is proportional to the amplitude of EMG signals whenever they are present. An advantage of continuous proportional myoelectric control is that it achieves patient-guided rehabilitation training which can improve patient participation during motion. A linear proportional model cannot consider subject-specific biomechanical factors during body movement, but an EMG-driven musculoskeletal model (EDMM) can enable more natural and human-like human-robot cooperation (Ao et al., 2017). Therefore, the EDMM has been widely used in controller design to detect the subject's effort (Walid et al., 2013; Ai et al., 2016).

Although patient participation has been investigated in previous studies (Song et al., 2013; Ai et al., 2016), the biomechanical properties of joint are subject-specific. However, these studies did not consider this characteristic, especially joint stiffness changes related to EMG activation and limb position (Kubo, 2014; Kung et al., 2015; Zhou et al., 2016). Thus, when the joint stiffness changes greatly, the control performance will be worse. Therefore, to achieve compliant assistance, the parameters of the inner position loop should be adjusted to suit the changes in joint stiffness. Previous studies have presented model-based approaches to estimate joint stiffness accurately, such as an EDMM (Pfeifer et al., 2012; Sartori et al., 2015).

Although there are advantages in admittance control and EDMM, few studies have combined these two methods into a control scheme. To encourage voluntary participation and realize compliant assistance, an EDMM that can estimate ankle joint torque and stiffness was applied in this study. In the proposed adaptive admittance control scheme (AACS), an admittance filter is applied to generate the intended motion based on the estimated joint torque. The parameters of the inner PD position loop are adjusted in real time according to the estimated joint stiffness and applied to determine the assistive torque. In the non-adaptive admittance control scheme (NAACS), the parameters of the inner position controller were set at four different levels. To evaluate the performance of the control schemes, the jerk value and position error derived from the tracking trajectory were applied to assess the smoothness and accuracy. The interaction torque and EMG levels of the tibialis anterior (TA) and gastrocnemius (GAS) were applied to quantitatively analyze muscular effort.

## Methods

### Ankle exoskeleton

**Figure 1:**
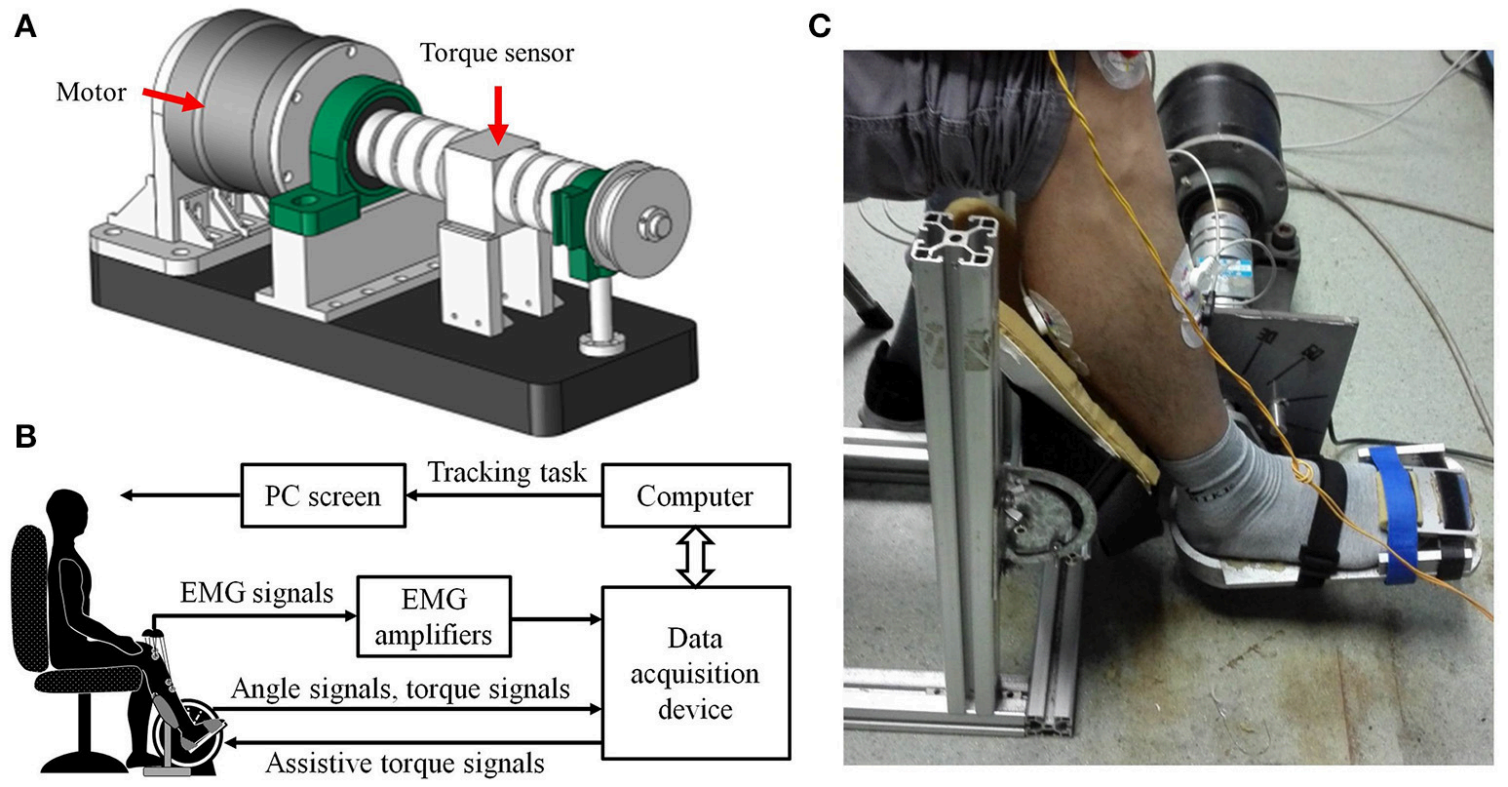
The ankle rehabilitation system. **(A)** The CAD graph of the ankle exoskeleton; **(B)** Schematic diagram of experimental apparatus. **(C)** The photo of the experiment setup.

The hardware of the ankle exoskeleton system mainly comprises a direct-drive (DDR) brushless AC servo motor (DM1B-045G, Yokogawa, Japan) with a servo driver (UB1DG3, Yokogawa, Japan), controller (GTS-400-PV(G)-PCI, Googoltech, Hong Kong), a torque sensor (AKC-205, 701st Research Institute of China Aerospace, Science and Technology Corporation, China), a personal computer (PC) with a screen, a PC-based data-acquisition device (USB-6341 DAQ card, National Instruments, USA), two custom-designed EMG amplifiers, and a mechanical footplate and supporter. The mechanical structure of the ankle exoskeleton is shown in Figure 1A. The motor was fixed to the ground and can rotate the footplate to a certain angle. The internal encoder can measure the rotation angle. The ends of the torque sensor were connected to the motor and the footplate. A supporter was applied to fix the position of the lower limb. The software of the ankle exoskeleton system mainly includes a Labview-based program (National Instruments, USA) and MATLAB code (MatlabR2014a, MathWorks Inc., Natick, MA, USA). The Labview-based program was applied to store the signals and calculate the assistive torque. The MATLAB code was applied to calibrate the EDMM and off-line analysis.

A diagram of the ankle exoskeleton system is shown in Figure 1B. The EMG of the TA and GAS was captured using EMG electrodes (Noraxon, Scottsdale, USA) and amplified by a factor of 5,000 by the EMG amplifiers. Then the EMG, angle, and torque signals were collected on the DAQ card, input to the PC, and stored by the Labview program for off-line analysis. The assistive torque signals were generated by the Labview program based on the control scheme and input to the motor through the DAQ card to provide the assistive torque. The PC screen was placed in front of the subject and used to provide visual feedback by displaying the target and the actual joint angle used for the tracking task.

### EMG-driven musculoskeletal model

The EDMM was first developed by Hoy et al. for computer simulation studies of musculotendon function and muscle coordination during movement (Hoy et al., 1990). Then, the study indicated that the EDMM is a good way to estimate *in vivo* muscle forces during movement tasks (Lloyd and Besier, 2003). Based on these studies and our previous study (Ao et al., 2017), an EDMM of the ankle joint was applied to calculate the joint torque. Since dorsiflexion was performed when the robot controlled by AACS or NAACS, only the EDMM of TA, the main dorsiflexor of ankle joint, was included. The EDMM mainly consists of three sub-models: an EMG-to-activation sub-model, a musculoskeletal geometry sub-model, and a Hill-based muscle-tendon sub-model. The EMG-to-activation sub-model was used to calculate the level of muscle activation using the raw EMG signals. The musculoskeletal geometry sub-model was used to calculate the length *l*_*mt*_ and torque arm *r*_*mt*_ of the muscle-tendon unit. And the Hill-based muscle-tendon sub-model was applied to predict the muscle-tendon forces.

In EMG-to-activation sub-model, the raw EMG signals were band-pass filtered between 20 and 450 Hz using a fourth-order Butterworth filter and then normalized with respect to the muscle's maximum voluntary contraction (MVC) level (Canning et al., 2000). After full-wave rectification of the normalized EMG signals, a low-pass second-order Butterworth filter at 2 Hz was applied to obtain the envelopes of the EMG signals (Canning et al., 2000). A recursive filter proposed by Lloyd and Besier (2003) was then used to calculate the neural activation *u*(*t*) from the processed signal *e*(*t*) using the following equation:

(1) $$\begin{array}{l} u\left(t\right)=\alpha e\left(t-d\right)-\beta_{1}u\left(t-1\right)-\beta_{2}u\left(t-2\right)\\ \left(\alpha =0.9486,\beta_{1}=-0.052,\beta_{2}=0.000627\right), \end{array}$$

where *d* is the electromechanical delay, which was set to 80 ms in this study (Lloyd and Buchanan, 1996). Since there is a non-linear relationship between the neural activation and muscle contraction force at low level of force (Manal and Buchanan, 2003), the relationship between the neural activation *u*(*t*) and muscle activation *a*(*t*) is expressed as

(2) $$\begin{array}{l} \{\begin{array}{ll} a(t)=b\ln(cu(t)+1), & 0\leq u(t)<u_{0}\\ a(t)=mu(t)+n, & u_{0}\leq u(t)<1. \end{array} \end{array}$$

In this curve, the node point (*u*_0_, *a*_0_) could be given as

(3) $$\begin{array}{l} \{\begin{array}{l} u_{0}=0.3085-A\cos(45{}^{\circ})\\ a_{0}=0.3085+A\sin(45{}^{\circ}). \end{array} \end{array}$$

The coefficients *m* and *n* can be calculated by knowing that the curve must pass through the node point (*u*_0_, *a*_0_) and (1, 1). Since the derivative of the linear and non-linear portions of the curve is equally, the value of *b* is iteratively obtained using the Newton–Raphson method and *c* was determined by Equation (4). Hence, the coefficients *b, c, m*, and *n* can be easily determined by knowing the node point (*u*_0_, *a*_0_) determined by *A* which would be determined during model calibration.

(4) $$\begin{array}{l} c=\frac{e^{a_{0}/b}-1}{u_{0}}. \end{array}$$

In musculoskeletal geometry sub-model, since the TA spans only one ankle joint and no wrapping points or wrapping surfaces associated with TA was assumed in this study (Schutte, 1993), the muscle-tendon unit length was viewed to be a straight-line path. Then, the length *l*_*mt*_ and torque arm *r*_*mt*_ of the muscle-tendon unit were calculated by the following equations (An et al., 1984; Feng et al., 1999):

(5) $$\begin{array}{l} l_{mt}=\sqrt{OA^{2}+OB^{2}-2OA*OB*\cos q}\\ =l_{t}+l_{m}\cos\phi , \end{array}$$

(6) $$\begin{array}{l} r_{mt}=\frac{\partial l_{mt}}{\partial q}, \end{array}$$

where *A* is the origin point, *B* is the insertion point, *O* is the center of the joint, *q* is the joint angle, *l*_*t*_ is the tendon length, *l*_*m*_ is the muscle fiber length, and ϕ is the pennation angle which can be given by

(7) $$\begin{array}{l} \phi =\sin^{-1}\frac{l_{0}\sin\phi_{0}}{l_{m}}, \end{array}$$

where *l*_0_ is the optimum fiber length and ϕ_0_ is the optimal pennation angle.

**Figure 2:**
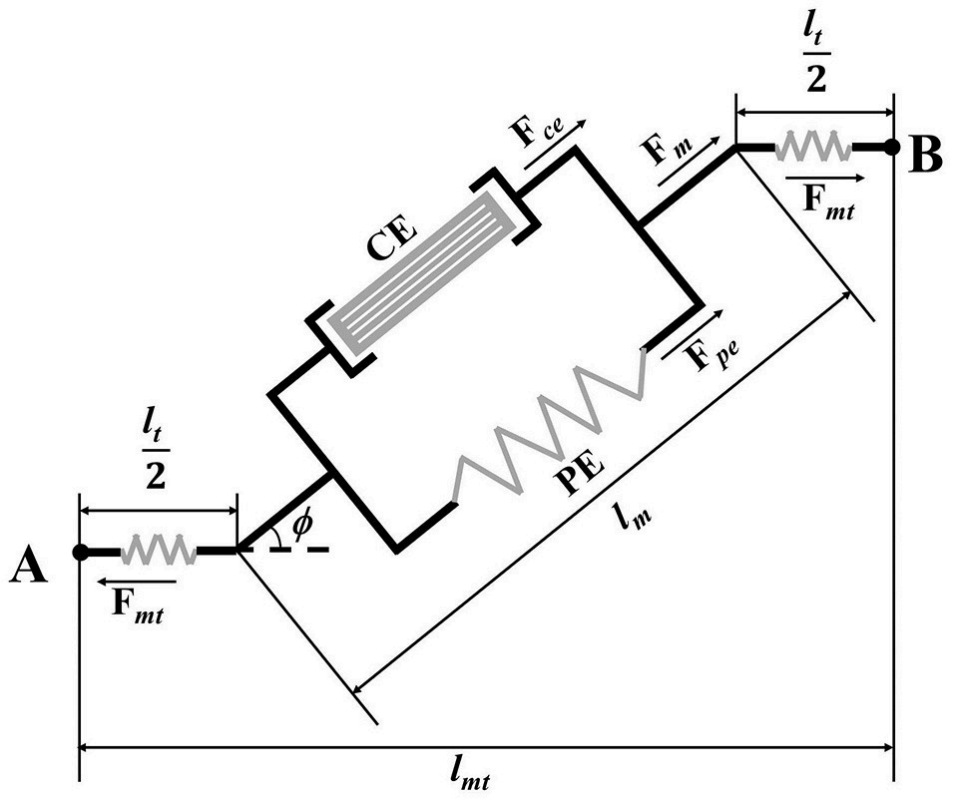
Schematic of Hill-based muscle-tendon model.

The Hill-based muscle-tendon sub-model consists of three parts: the contractile element, the passive element, and the tendon (Hill, 1938; Hoy et al., 1990). The contractile element of muscle fibers can actively generate force *F*_*ce*_. The passive element of muscle fibers is parallel to the active components, as shown in Figure 2. And the tendon can be modeled as a non-linear spring. The force produced by the muscle-tendon unit *F*_*mt*_ is expressed as

(8) $$\begin{array}{l} F_{mt}=F_{m}\cos\phi =\left(F_{ce}+F_{pe}\right)\cos\phi =F_{t}, \end{array}$$

where *F*_*m*_, *F*_*ce*_, *F*_*pe*_, and *F*_*t*_ represent the force generated by the muscle fibers, contractile element, passive element and tendon, respectively.

The force *F*_*ce*_ actively generated by contractile element of muscle fibers is related to the magnitude of the muscle activation input *a* and maximum isometric muscle force *F*_*mx*_:

(9) $$\begin{array}{l} F_{ce}=f\left(l\right)f\left(v\right)F_{mx}a, \end{array}$$

where *f*(*l*) and *f*(*v*) are the active force–length relationship (Giat et al., 1994) and force–velocity relationship (Schutte, 1993) as defined by Equations (10, 11), respectively; *l* is the normalized muscle fiber length, which is the ratio of the current muscle fiber length *l*_*m*_ to the optimum fiber length *l*_*m*0_ at activation *a*; *v* is the ratio of the current muscle-fiber velocity to the maximum contraction velocity. The current muscle-fiber velocity is the derivative of the length of muscle fiber and the maximum contraction velocity is set to 10*l*_0_/s (Zajac, 1989) in this study.

(10) $$\begin{array}{l} f\left(l\right)=\sin\left(-1.317l^{2}-0.403l+2.454\right), \end{array}$$

(11) $$\begin{array}{l} \{\begin{array}{ll} f(v)=\frac{0.3(v+1)}{-v+0.3} & v<0\\ f(v)=\frac{2.34v+0.039}{1.3v+0.039} & v>0 \end{array} \end{array}$$

To reduce the impact of muscle activation on the optimal fiber length, Equation (12) is applied.

(12) $$\begin{array}{l} l_{m0}=l_{0}\left(\lambda \left(1-a\right)+1\right), \end{array}$$

where the λ denotes the percentage of change in the optimal fiber length, which was determined to be 0.15 (Lloyd and Besier, 2003).

The corresponding passive forces *F*_*pe*_ can be expressed as:

(13) $$\begin{array}{l} F_{pe}=f_{p}\left(l\right)F_{mx}, \end{array}$$

where *f*_*p*_(*l*) is the passive elastic force–length relationship (Buchanan et al., 2004), as defined by

(14) $$\begin{array}{l} f_{p}\left(l\right)=0.129\left(e^{4.525\left(l-1\right)}-1\right). \end{array}$$

The magnitude of the tendon contraction and its force *F*_*t*_ is related to the tendon's current length *l*_*t*_ and its slack length *l*_*st*_ (Zajac, 1989):

(15) $$\begin{array}{l} F_{t}=\{\begin{array}{ll} 0 & \varepsilon \leq 0\\ 1480.3F_{mx}\varepsilon^{2} & 0<\varepsilon \leq 0.0127,\\ (37.5\varepsilon -0.2375)F_{mx} & \varepsilon \geq 0.0127 \end{array} \end{array}$$

$$\begin{array}{l} \varepsilon =\frac{l_{t}-l_{st}}{l_{st}}. \end{array}$$

The Runge-Kutta-Fehlberg algorithm was applied to solve the differential equation based on Equation (8) to obtain the length of muscle fiber, then the *F*_*mt*_ can be calculated using the length of muscle fiber according to Equation (8). Then corresponding joint torque *M*_*p*_ can be estimated by the product of the torque arm *r*_*mt*_ and force *F*_*mt*_:

(16) $$\begin{array}{l} M_{p}=r_{mt}F_{mt}. \end{array}$$

In this model, the unknown parameters of the muscle activation parameter *A*, the tendon slack length *l*_*st*_, and the maximum isometric muscle force *F*_*mx*_ were determined by minimizing Equation (17) according to the EMG and measured torque *M*_*m*_ in a MVC experiment, which is described in section Model Calibration. The MATLAB (MatlabR2014a, MathWorks Inc., Natick, MA, USA) and Nelder-Mead algorithm were applied to minimize the Equation (17):

(17) $$\begin{array}{l} Er=\frac{1}{N}\overset{N}{\underset{i=0}{\sum }}{\left(M_{p}\left(i\right)-M_{m}\left(i\right)\right)}^{2}, \end{array}$$

where *N* represents the length of the data used for the calibration. The other parameters (OA, OB, *l*_0_, and ϕ_0_) were assigned using OpenSim (National Institutes of Health for Biomedical Computation, Stanford, USA). The details of our EDMM can be found in Ao et al. (2017).

### Joint stiffness estimation

The muscle-tendon unit stiffness can be modeled as the muscle fiber stiffness *K*_*m*_ in series with the tendon stiffness *K*_*t*_ (Cui et al., 2008; Pfeifer et al., 2012). The model assumes that *K*_*m*_ is a function of the muscle force *F*_*m*_, the muscle-fiber length at maximum activation *l*_*m*0_, and a dimensionless scaling constant γ, which is 23.4 (Cui et al., 2008):

(18) $$\begin{array}{l} K_{m}=\frac{\gamma F_{m}}{l_{m0}}. \end{array}$$

In the model, *K*_*t*_ is defined by the slope of the generic, dimensionless force-strain curve scaled for each individual tendon (Zajac, 1989):

(19) $$\begin{array}{l} K_{t}=\frac{dF_{t}}{dl_{t}}. \end{array}$$

Therefore, the muscle-tendon unit stiffness can be obtained by

(20) $$\begin{array}{l} K_{mt}=\frac{K_{m}K_{t}}{K_{m}+K_{t}}. \end{array}$$

Using the estimated muscle forces and the muscle-tendon unit stiffness, we compute the corresponding joint stiffness *K*_*j*_, while considering the kinematic relationship between changes in joint angles and muscle-tendon length (Pfeifer et al., 2012).

(21) $$\begin{array}{l} K_{j}=K_{mt}r_{mt}^{2}+\frac{\partial r_{mt}}{\partial q}F_{mt}. \end{array}$$

### Adaptive admittance control scheme

To provide motion assistance, the patient's intended direction of motion is considered to be the direction of the estimated joint torque. When the patient's active joint torque is along a certain direction, the intended motion will be in the same direction. In this case, the admittance filter is appropriate, which can define any dynamic relationship between a position and force (Culmer et al., 2010):

(22) $$\begin{array}{l} q_{i}\left(s\right)=q\left(s\right)+\frac{M_{p}\left(s\right)}{k+bs+ms^{2}}, \end{array}$$

where *M*_*p*_ is the joint torque estimated by the EDMM; and *k, b*, and *m* are the stiffness, damping, and mass parameters, respectively, which can be determined empirically. Because the end-effector's acceleration was small in this study, the impact of the acceleration change could be ignored (Xu et al., 2011; Saglia et al., 2013), and *m* was set to 0. Also, *k* and *b* were set to 2 and 0.1, respectively. In this case, the tracking task can be done easily. For all subjects, the same parameter settings were applied.

A PD position controller was necessary to track the intended motion of the subject, which can be expressed by

(23) $$\begin{array}{l} \tau =K\Delta q+B\Delta \dot{q}, \end{array}$$

where Δ*q* is the error between the actual angle and the intended angle, and *B* is determined by the following equation (Liang et al., 2014, 2015):

(24) $$\begin{array}{l} B=0.2\sqrt{K}. \end{array}$$

**Figure 3:**
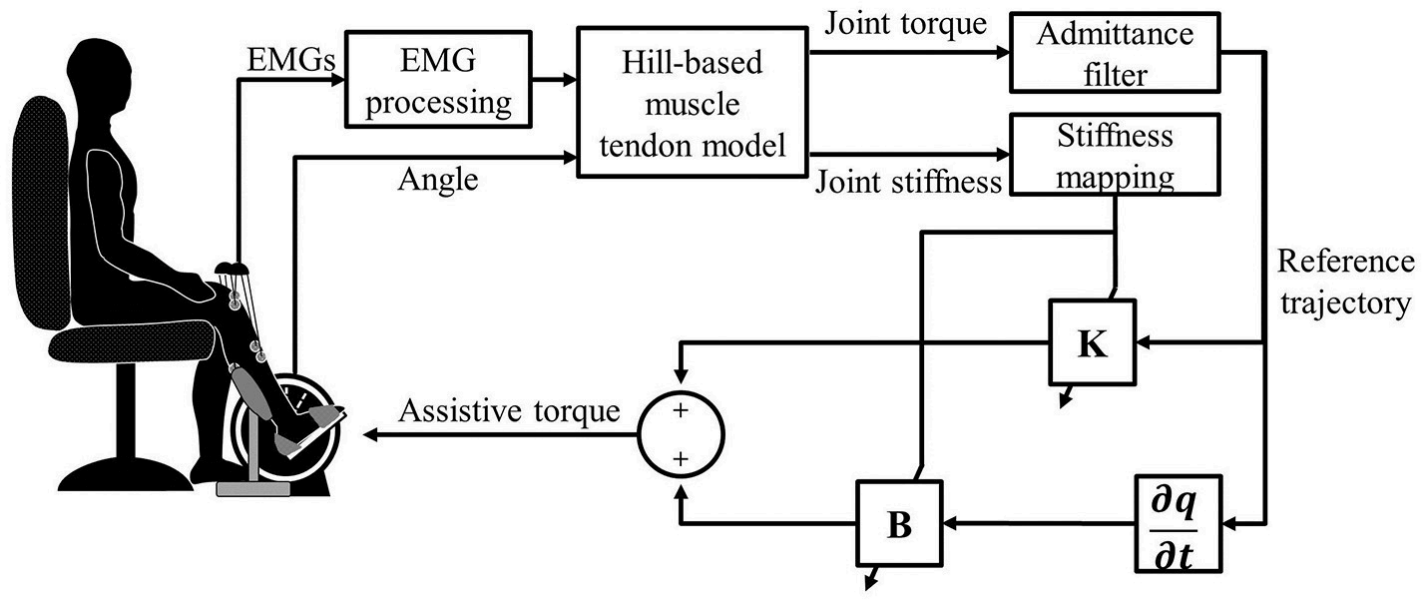
The control diagram of the ankle rehabilitation system.

The NAACS was designed using a fixed value of *K* in Equation (23). Also, *K* was empirically set to four levels (0, 0.1, 0.3, and 0.5) in this study. In contrast, the parameters *K* and *B* of the PD controller were adjusted in real time with consideration of the changes in the estimated joint stiffness during movement when the AACS was used. The controller boundaries of *K* for stable operation (*K*^max^ and *K*^min^) can be obtained experimentally (Liang et al., 2015). We used Equation (25) to incorporate these boundaries:

(25) $$\begin{array}{l} K=\left(K^{\max}-K^{\min}\right)\frac{K_{j}-K_{j}^{\min}}{K_{j}^{\max}-K_{j}^{\min}}+K^{\min}, \end{array}$$

where *K*_*j*_ is the joint stiffness based on estimation from EMG; $K_{j}^{\max}$ and $K_{j}^{\min}$ are, respectively, the maximum and minimum joint stiffness values predicted by the calibrated EDMM during tracking tasks without robot assistance; and *K* is the modified parameter value of the controller based on the joint stiffness. The structure of the AACS is shown in Figure 3.

## Experiments

### Experimental setup

A total of eight healthy subjects (two females and six males; age: 22–25 years) without any neurological or motor disorders participated in the study. Before participating in the experiment, all subjects were informed about the experimental procedures, potential risks and purpose of the experiment. Written informed consent was obtained from all the subjects, and this study was approved by the Human Ethic Committee of Sun Yat-sen University. The experimental setup is shown in Figure 1C. All subjects were asked to be seated in a chair and place their feet on the footplate with a supporter fixing the right foot. To measure EMG signals, two pairs of circular electrodes (Ag-AgCl, 1-cm diameter) were attached to the skin surface at the centers of the muscle bellies of the TA and GAS with a center-to-center distance of 2 cm along the longitudinal axis (Sun et al., 2016). The EMG signals were sampled by the DAQ at a sample rate of 1,000 Hz and then band-pass filtered from 20 to 450 Hz, full-wave rectified, and low-pass filtered at 2 Hz (Canning et al., 2000). A computer screen was placed in front of the subjects to offer guidance information for visual feedback (EMG signals, torque signals, and an indicator light).

### Model calibration

Since on-line control using the EDMM is applied after off-line calibration, to balance calibration time and predict accuracy of EDMM, the three parameters were calibrated by the method reported in Fleischer et al.'s study (Fleischer and Hommel, 2008). In this study, MVC experiments were conducted with the ankle joint of each subject positioned at 90 and 120° in the vertical plane. At the beginning of the experiments, the subjects were asked to relax all the muscles in the ankle joint as much as possible. Then, the TA was activated by the subjects as much as possible when the indicator light turned on until it turned off again. The duration of each trial in the experiments was 5 s, and three trials were performed for each angle. The maximal EMG amplitude of the TA at each angle was recorded during the experiments and stored to calibrate the EDMM.

### Stiffness verification

After the EDMM was calibrated using the MVC data, the joint torque and joint stiffness could be estimated in real time. Then, perturbation tasks were performed to verify the accuracy of the estimated joint stiffness. A healthy subject's foot was placed on the foot-pedal, and the ankle angle was 90° with relaxed muscles. The subject received information from the indicator light as well as information about the degree of TA activation from the screen.

During the experiment, each subject was asked to maintain certain activation levels of the TA when the indicator light was on (10, 15, 20, 25, 30, and 35% MVC). A series of perturbations were applied by the motor, which was controlled by the PD controller (*P* = 5, *D* = 0.5). The desired trajectory of the motor was sinusoidal with an amplitude of 0.5° and a frequency of 3 Hz. The duration of each trial of each activation level of the TA was 30 s, and two trials were performed for each activation level. The joint stiffness was measured using a multiple linear regression model based on the torque signals and position signals. The joint stiffness could also be estimated by the calibrated EDMM. To assess the accuracy of the ankle stiffness estimated by the EDMM, linear correlation analysis between the measured and estimated stiffness was conducted.

### Trajectory tracking task

Finally, a series of sinusoidal ankle tracking tasks were performed to assess the performance of different controllers. Each subject was asked to sit in a chair in the same posture adopted during the MVC experiments. The computer screen displayed a blue slider for the target angle and a red slider for the actual ankle angle. It should be noted that the target angle of the sinusoidal trajectory was only used to provide visual guidance, and the assist torque only related to the subject's intention position and joint stiffness. In each trial, the initial position of the ankle joint was set at 90°, and after a random delay of 3–5 s, the target slider would move along a preset sinusoidal trajectory with an amplitude of −25 to 25°. Eleven cycles of sinusoidal tracking movements were conducted in each trial, which took 110 s.

After one or two practice trials, the first trial was performed, in which the robot was controlled by the NAACS and *K* was set to 0. The mapping rules of the AACS were determined according to the variation of the actual ankle joint stiffness during the first trial. Then, the AACS and NAACS were randomly selected in the following trials, and each control scheme was repeatedly selected two times. The subjects were blinded to the type of controller in each trial, and only dorsiflexion was performed using the control scheme to assist subjects in following the target slider. During the experiment, the subjects were asked to track the target slider as accurately as possible and could take breaks when they felt tired.

### Signal processing and statistical analysis

In the tracking task, when a subject moved to nearly −25 or 25°, the speed of tracking was equal to zero to change the direction of movement. At this time, dynamic friction in the system disappeared, and static friction was dominant. Generally, static friction is much stronger than dynamic friction, and it is difficult to compensate static friction force in the output torque. To deal with static friction when the movement changed direction, subjects tended to reduce their effort to track the trajectory in varying degrees. Therefore, to eliminate the impact of static friction on the tracking task, experimental data from only the movement range of −20 to 20° were used in the analysis. To evaluate the performance of the control strategies, the root-mean-squared jerk was considered as an effective measurement of the movement smoothness. Smaller values of jerk-based indexes indicate higher smoothness (Hogan and Sternad, 2009), which can be expressed by

(26) $$\begin{array}{l} Jerk\;value=\sqrt{\frac{1}{N}\overset{N}{\underset{i=1}{\sum }}J{\left(i\right)}^{2}}, \end{array}$$

where *J*(*i*) is calculated from the third derivative of the actual angle at the *i*th sampling instant.

The root-mean-squared error between the actual and desired joint positions was used to evaluate the tracking accuracy of the controller:

(27) $$\begin{array}{l} Position\;error=\sqrt{\frac{1}{N}\overset{N}{\underset{i=1}{\sum }}{\left(q_{d}\left(i\right)-q\left(i\right)\right)}^{2}}. \end{array}$$

The root-mean-squared value was used for the interaction torque to demonstrate its magnitude during the ankle tracking tasks. The root-mean-squared value was also used for the EMG signals from the TA and GAS to evaluate muscular effort, which can be determined as follows:

(28) $$\begin{array}{l} Interaction\;torque=\sqrt{\frac{1}{N}\overset{N}{\underset{i=1}{\sum }}\tau {\left(i\right)}^{2}}, \end{array}$$

(29) $$\begin{array}{l} EMG\;level=\sqrt{\frac{1}{N}\overset{N}{\underset{i=1}{\sum }}EMG{\left(i\right)}^{2}}. \end{array}$$

The indicators were analyzed off-line using MATLAB (MatlabR2014a, MathWorks Inc., Natick, MA, USA). Correlation analysis between the measured stiffness and estimated stiffness was carried out using Microsoft Excel 2010. Statistical analysis was conducted using SPSS 20.0 (SPSS Inc., Chicago, IL, USA), and one-way analysis of variance (ANOVA) with repeated measures was applied to compare every indicator of the control schemes. In addition, a *post-hoc* analysis using the least-significant difference was carried out when there was a significant difference between the considered control schemes. A significance level of 0.05 was set for all statistical tests.

## Results

**Figure 4:**
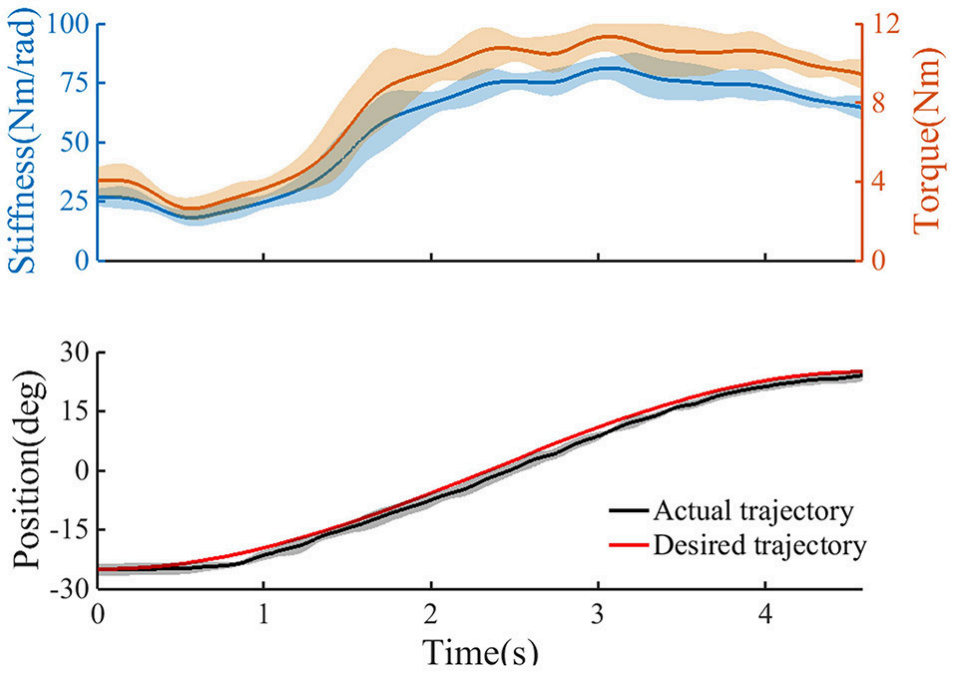
The trajectory tracking task without provided assistance (*n* = 2 trials); **Top**: the estimated user's torque, and the estimated joint stiffness. **Bottom**: the ankle angle as recorded from the device. Continuous line, average curves; shaded area, standard deviation.

Table 1 gives the results of the measured and estimated stiffness from one subject at various activation levels of the TA. The results of the correlation analysis show that there was a significant correlation between the measured and estimated stiffness (*R* = 0.992). Figure 4 shows the change in estimated joint torque and joint stiffness during trajectory tracking task without provided assistance.

**Table 1:** Measured and estimated joint stiffness (Nm/Rad) of one subject at different activation levels of TA.

	**MVC%**						
	0	10	15	20	25	30	35
Measured	15.18	28.20	39.58	46.20	54.84	59.88	71.07
Estimated	14.19	34.49	46.37	53.50	59.40	65.41	75.51

**Figure 5:**
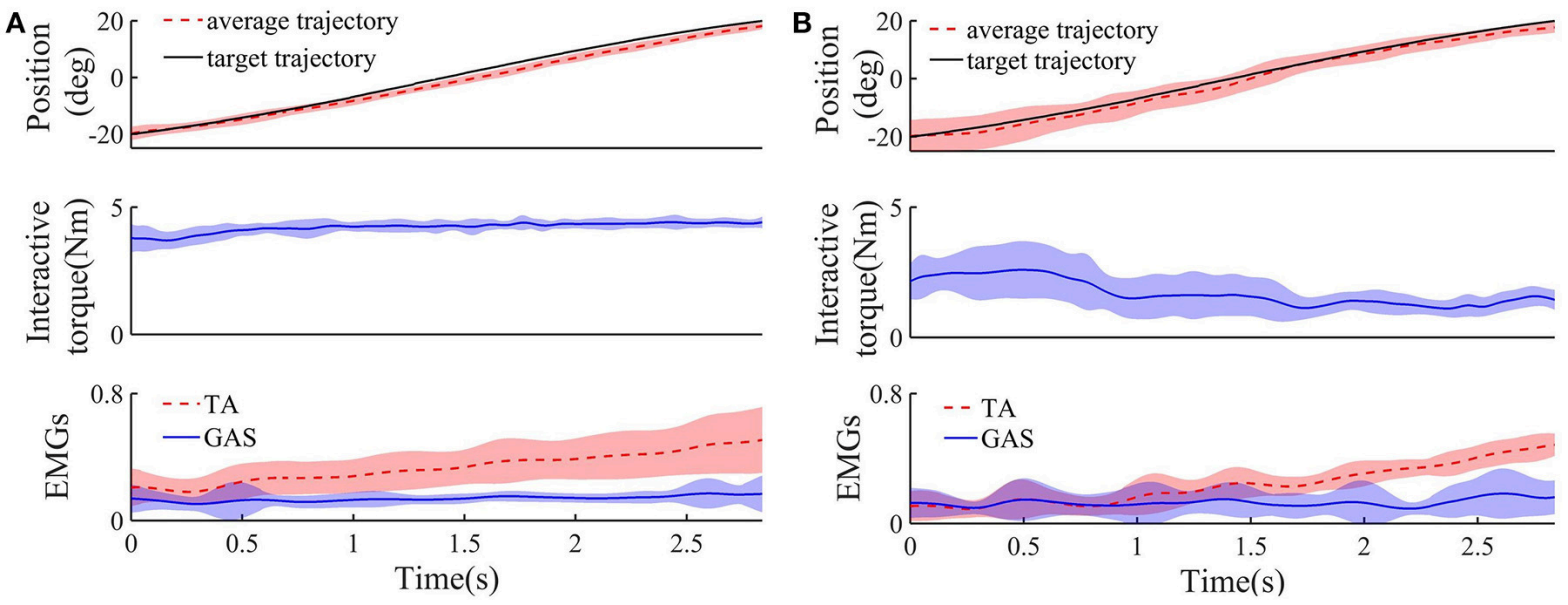
The target, average tracking trajectory, average interaction torque, and average processed EMG signals from TA and GAS of all subject during dorsiflexion phase when robot was controlled by NAACS and K was set at 0 **(A)** and 0. 5 **(B)**, respectively (*n* = 16 trials). Solid or dotted line, average curves; shaded area, standard deviation.

**Figure 6:**
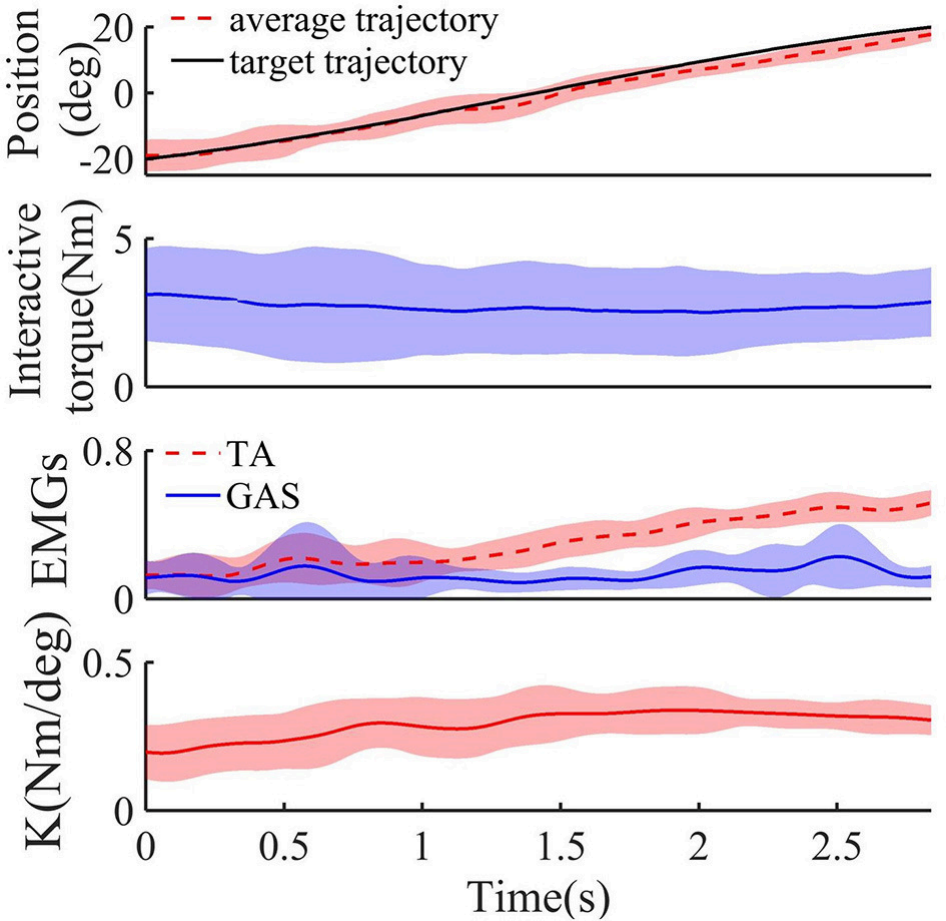
The target, average tracking trajectory, average interaction torque, average processed EMG signals from TA and GAS, and average *K*-value of all subject during dorsiflexion phase when robot was controlled by AACS (*n* = 16 trials). Solid or dotted line, average curves; shaded area, standard deviation.

Figure 5 shows the target and average tracking trajectories, average interaction torque, and average processed EMG signals from the TA and GAS during the tracking task when the robot was controlled by the NAACS. The *K*-value of the NAACS is 0 in Figure 5A and 0.5 in Figure 5B. Figure 6 shows the results of the target and average tracking trajectories, average interaction torque, average processed EMG signals from the TA and GAS, and average *K*-value during the tracking task when the robot was controlled by the AACS.

**Figure 7:**
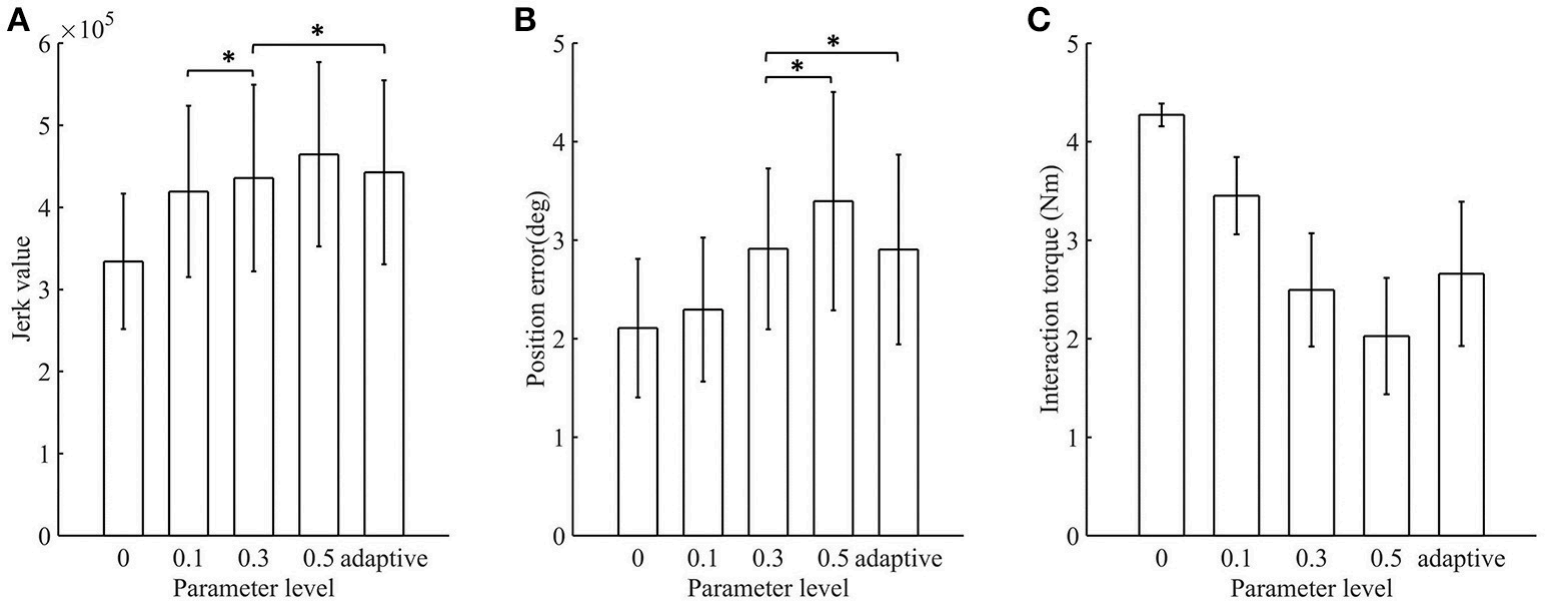
The mean value and standard deviation of indicators at different control schemes: **(A)** Jerk value; **(B)** Position error; **(C)** Interaction torque. Adaptive, AACS. ^*^indicates that there was no significant difference between the groups (*p* > 0.05 and *n* = 80 trials).

The jerk values of the AACS and NAACS are shown in Figure 7A. For the NAACS, there was a significant upward trend as *K* increased (*P* < 0.01). There was also a significant difference between the jerk values of *K* of 0 and 0.1 (*P* < 0.01). A significant difference also can be found between the *K*-values of 0.3 and 0.5 (*P* < 0.01). However, the increase was not significant between *K*-values of 0.1 and 0.3 (*P* = 0.100). In addition, when *K* was set to 0.5, there was a significant increase in comparison with *K* of 0.1 (*P* = 0.002).

When the AACS was applied, the jerk values were higher than those of the NAACS when *K* was set to 0, 0.1, and 0.3. However, they were lower than those of the NAACS when *K* was set to 0.5, as seen in Table 2. There was no significant difference between the jerk values between of the AACS and NAACS when *K* was set to 0.3 (*P* = 0.241).

**Table 2:** The mean (standard deviation) of jerk value, position error, interaction torque, TA EMG level, GAS EMG level under different control schemes (*n* = 80 trials).

**Indicator**	**Control scheme**				
	**NAACS**				**AACS**
	*K* = 0	*K* = 0.1	*K* = 0.3	*K* = 0.5	
Jerk Value(×105)	3.34 (0.83)	4.19 (1.04)	4.36 (1.14)	4.65 (1.12)	4.43 (1.12)
Position error (deg)	2.11 (0.70)	2.30 (0.73)	2.91 (0.82)	3.40 (1.11)	2.91 (0.96)
Interaction torque (Nm)	4.27 (0.12)	3.45 (0.39)	2.50 (0.58)	2.03 (0.59)	2.66 (0.73)
TA EMG level (×100%)	0.31 (0.11)	0.27 (0.09)	0.24 (0.09)	0.21 (0.07)	0.24 (0.08)
GAS EMG level (×100%)	0.14 (0.05)	0.13 (0.04)	0.13 (0.05)	0.13 (0.04)	0.12 (0.04)

The position errors of the AACS and NAACS are shown in Figure 7B. There was a significant upward trend as *K* increased for the NAACS (*P* < 0.01). The difference between *K* of 0 and 0.1 was significant (*P* = 0.047), as well as that between *K* of 0.1 and 0.5 (*P* = 0.02). However, there was no significant difference between *K* of 0.3 and 0.5 (*P* = 0.123). As given in Table 2, the position error of the AACS was equal to that of the NAACS when *K* was set to 0.3. The difference between them was not significant (*P* = 0.958). However, there were significant differences between the AACS and NAACS when *K* was set to 0 (*P* = 0.029), 0.1 (*P* = 0.031), and 0.5 (*P* = 0.031).

The interaction torques of the AACS and NAACS are shown in Figure 7C. For the NAACS, there was a significant downward trend as the *K* increased (*P* < 0.01). Significant differences were found between each two successive *K*-values (0–0.1, *P* < 0.01; 0.1–0.3, *P* < 0.01; 0.3–0.5, and *P* = 0.001). For the AACS, the interaction torque was higher than that of the NAACS when *K* was set to 0.3 and 0.5, while it was lower than that of the NAACS when *K* was set to 0 and 0.1. The difference was significant between the AACS and NAACS when *K* was set to 0 (*P* < 0.01), 0.1 (*P* = 0.02), 0.3 (*P* = 0.032), and 0.5 (*P* = 0.001).

**Figure 8:**
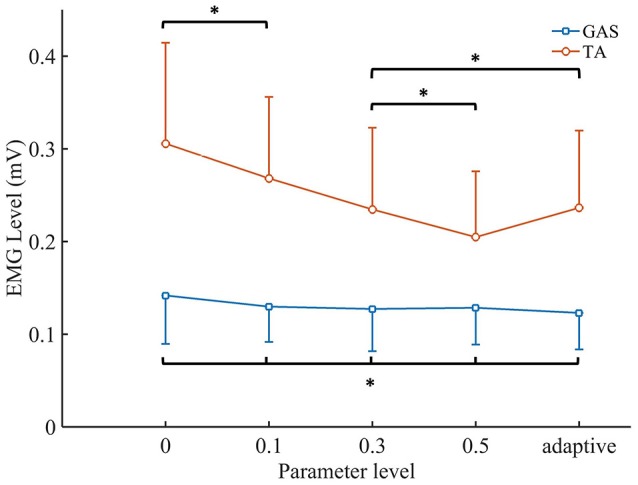
The mean values and standard deviation of TA's EMG level and GAS's EMG level. Adaptive, AACS. ^*^indicates that there was no significant difference between the groups (*p* > 0.05 and *n* = 80 trials).

The EMG levels of the TA and GAS are shown in Figure 8. For the NAACS, the EMG levels of the TA showed a significant downward trend as *K* increased (*P* < 0.01). Significant differences were found between several *K*-values (0.1–0.3, *P* = 0.018; 0–0.3, *P* = 0.03; 0.1–0.5, *P* < 0.01). However, there was no significant difference between *K* of 0 and 0.1 (*P* = 0.056) nor between *K* of 0.3 and 0.5 (*P* = 0.072). For the AACS, the EMG levels of the TA were equal to that of the NAACS when *K* was set to 0.3, and there was no significant difference between them (*P* = 0.825). When *K* was set to 0, 0.1, and 0.5, there were significant differences between the NAACS and AACS (*P* = 0.005, *P* = 0.003, and *P* = 0.004, respectively). The EMG levels of the GAS clearly showed no significant differences between the AACS and NAACS nor between NAACS when *K* was set to various levels (*P* = 0.250).

## Discussion

### Joint stiffness estimation based on the EMG signals

In previous studies, joint stiffness was mainly obtained by experimental measurements such as rapid ramp and hold perturbations (Cui et al., 2008; Guarin and Kearney, 2015; Lee and Hogan, 2015). For example, Mussa-Ivaldi et al. developed a perturbation method to measure hand stiffness using a manipulator to displace the subject's hand during maintenance of a given posture (Mussa-Ivaldi et al., 1985). Although above studies have explored how to measure or estimate joint stiffness through offline analysis except for the model-based method developed and verified by Pfeifer et al. (2012), the dynamic and continuous changes in joint stiffness have not received much attention. In this study, joint stiffness was estimated and recorded in real time based on EDMM during tracking task. At the beginning of a movement, a phenomenon can be observed is that the joint stiffness and torque were relatively low. This indicates that trajectory tracking did not require the subject to maintain a large joint stiffness and torque when the actual position was very close to the target position and the movement speed was lower. However, when the subject tried to track the target position quickly and accurately by activating the TA, a rapid rise in joint stiffness and torque occurred. In the final phase of dorsiflexion, joint stiffness and torque tended to decline slowly owing to the slower speed of the tracking target. However, the joint stiffness and torque remained at high levels owing to the smaller dorsiflexion angle, even though the task had been completed. These trends of ankle joint stiffness and torque are similar to those observed in a previous study, which investigated changes in knee joint stiffness and torque during standing tasks (Karavas et al., 2014).

### Comparison between AACS and NAACS

For the NAACS, there was an increasing trend in the jerk value and position error as *K* increased, which demonstrates that subjects had difficulties in accurately and smoothly tracking a target controlled by the NAACS when *K* was set to a higher value. In particular, the jerk value and position error were the smallest when *K* was set to 0. This may be explained by the fact that the tracking performance of an ankle exoskeleton is not as good as that of a healthy subject, so a subject is able to track a target well when the ankle exoskeleton does not provide assistive torque. In a previous study, Kwon et al. (2014) investigated the variation in human movement stability while varying the assistive torque provided by a robotic device based on the EMG amplitude. They found that movements became unstable because of an increased assistive torque. The trends of the jerk values and position error with respect to *K* were consistent with this finding. Meanwhile, when *K* was set to 0, the subjects had to drive the ankle exoskeleton by their own efforts to complete the tracking task which results in a higher interaction torque. The interaction torque indicated the compliance of human-robot interaction, and higher torque could cause secondary damage to patients in rehabilitation training (Jamwal et al., 2016). Therefore, *K* of the NAACS should not be set at a lower value.

The EMG levels of the TA representing the muscle effort of subjects in tracking tasks decreased as *K* increased. Greater assistive torque was applied to the subjects when *K* was set to a higher level, and most of the working load was borne by the device (Lenzi et al., 2012; Ao et al., 2017). With higher K, the NAACS can effectively reduce the required muscle effort and make it easier for subjects to complete the training. It is worth noting that the EMG levels of the GAS showed no significant change, which can be explained by the GAS not being a dominant muscle in this task.

For the NAACS, when lower jerk value and position error were achieved, the interaction torque and EMG of the TA increased, and vice versa. Generally, excessive jerk, position error, interaction torque, or EMG levels are not expected to occur in rehabilitation training. When *K* was set to 0, 0.1, and 0.5, each indicator of the AACS was lower than the corresponding maximum value of the NAACS. Therefore, the AACS achieved a balance among all indicators (jerk value, position error, interaction torque, and EMG levels of the TA). This also ensured that the value of each indicator could not be too large. Similarly, Ajoudani et al. proposed a concept of tele-impedance control, in which the user's stiffness references are mapped to a robotic hand. Compared with constantly high or constantly low stiffness values, the tele-impedance control appears to strike a good compromise between the two extremes, which is consistent with the conclusions of this study (Ajoudani et al., 2016). In this study, although no significant difference was found between the AACS and NAACS with *K* = 0.3, the AACS still showed more satisfactory tracking performance in comparison to the NAACS with other *K*-values. Actually, when the position error is smaller, the interaction torque is larger for the NAACS, and vice versa. Therefore, a suitable *K*-value may be found for a specific subject or task, but a constant *K*-value should not be applied for all subjects owing to individual differences among subjects. Therefore, one advantage of the AACS in comparison to the NAACS is its ability to adaptively adjust the parameters of the inner position controller according to the joint stiffness.

### Implications for clinical applications

In the AACS, EMG signals that reflect the activities of a subject's muscles and angle signals are input into the EDMM to accurately estimate the active torque. Then, the intended motion is obtained by the admittance filter according to joint torque which was changing in real time. Therefore, the AACS can improve a subject's voluntary participation because the movement trajectory is determined by the subject rather than by a predefined program in PD or PID control. Furthermore, the AACS provides assistive torque through the inner position loop rather than the proportional myoelectric or estimated joint torque. Therefore, compared with conventional EMG control (Song et al., 2013; Kwon et al., 2014), the PD controller of the AACS can help improve the robustness and stability properties. The AACS can also allow a robot to be controlled in a more natural and human-like way by adjusting the parameters of the inner position loop according to the joint impedance.

In the previous study, the EDMM was used to estimate muscle forces and joint moments in stroke patients and the model did predict the ankle moment with acceptable accuracy (Shao et al., 2009). Although the performance of the AACS is verified on healthy subjects, there are still some limitations in this study. To improve the dynamic characteristics of the ankle exoskeleton, the static friction will be modeled and compensated in the future work. And the joint torque and stiffness will be estimated by using multiple muscles associated with the ankle joint (TA, GAS, soleus, etc.) to improve the performance of the AACS. In addition, to apply the AACS for the active rehabilitation of patients with motor disorders, the study on tuning of parameters in admittance filter will be carried out.

## Conclusion

This paper proposed an AACS in which the intended motion is determined by an admittance filter according to the joint torque. The parameters of the inner position controller were adjusted based on the joint stiffness. Compared with the NAACS, the AACS could balance accurate tracking with compliant assistance and control the robot in a way that combines the patient's intention and limb condition. The AACS has the potential to be applied in the rehabilitation training of stroke or spinal cord injury patients.

## Author contributions

SY and RS conceived and designed the study. SY and YZ performed the experiments. SY and RS wrote the paper. RS reviewed and edited the manuscript. ZL made a contribution to the edition of the manuscript. All authors had read and approved the manuscript.

### Conflict of interest statement

The authors declare that the research was conducted in the absence of any commercial or financial relationships that could be construed as a potential conflict of interest. The reviewer RT and handling Editor declared their shared affiliation.
